# 569. Characterization of COVID-19 Vaccine Breakthrough Infections in Metropolitan Detroit

**DOI:** 10.1093/ofid/ofab466.767

**Published:** 2021-12-04

**Authors:** Nicholas sturla, Rita Kassab, Rafa Khansa, Thomas Chevalier, David Allard, Robert Tibbetts, Linoj Samuel, Geehan Suleyman

**Affiliations:** 1 Henry Ford Hospital, Detroit, MI; 2 Henry Ford Health System, Detroit, MI

## Abstract

**Background:**

Although COVID-19 vaccines are very effective, vaccine breakthrough infections have been reported, albeit rarely. When they do occur, people generally have milder COVID-19 illness compared to unvaccinated people. A total of 10,262 (0.01%) SARS-CoV-2 vaccine breakthrough infections had been reported as of April 30, 2021. The objective of this study was to evaluate the effectiveness of COVID-19 vaccines and characterize breakthrough infections in our patient population.

**Methods:**

This was a retrospective review of all consecutive COVID-19 vaccine breakthrough infections at Henry Ford Health System (HFHS) in metropolitan Detroit, Michigan, from December 17, 2020 to June 7, 2021. Centers for Disease Control (CDC)'s breakthrough infection definition (detection of SARS-CoV-2 RNA or antigen in a respiratory sample ≥14 days after completion all recommended doses of COVID-19 vaccine) was used to identify cases. Vaccination status was extracted from the electronic medical records using Epic™ SlicerDicer.

**Results:**

A total of 228,674 patients, including healthcare workers (HCW), were fully vaccinated in our healthcare system. We evaluate 299 patients for breakthrough infection but only 179 (0.08%) patients met the definition; 108 (60%) were female with median age of 59, 60 (33%) were HCW, and 11 (6%) were immunocompromised. The majority (92%) were asymptomatic (62 or 35%) or had mild/moderate illness (102 or 57%); 14 (8%) had severe or critical illness. The status of one patient was unknown. Of those who were symptomatic, 24 (13%) required hospitalization, and 3 (2%) required intensive unit care. One patient admitted for heart failure exacerbation died unexpectedly prior to being discharged. Nine had previous COVID-19 within 4 months but only one was symptomatic; this likely represented residual shedding in the asymptomatic patients.

**Conclusion:**

COVID-19 vaccine was very effective among our patients and breakthrough infections were rare. Moreover, the vaccine reduced disease severity and mortality. Efforts should aim to increase vaccine uptake.

**Disclosures:**

**All Authors**: No reported disclosures

